# Joint Treatment of De Novo Umbilical Endometriosis with Plastic Surgery and Minimally Invasive Gynecologic Surgery

**DOI:** 10.1097/GOX.0000000000003675

**Published:** 2021-07-19

**Authors:** Angie Hamouie, Elizabeth Brunn, Joanna Orzel, Sarah R Shehr, James K Robinson

**Affiliations:** From the * National Center for Advanced Pelvic Surgery, Medstar Washington Hospital Center, Washington, DC; † Georgetown University School of Medicine, Washington, DC.

## INTRODUCTION

Endometriosis is a gynecological condition that affects 10%–15% of women of reproductive age.^1^ The disorder is characterized by ectopic implants of endometrial tissue, usually on pelvic reproductive organs.

The skin is an uncommon location for endometriosis. Cutaneous endometriosis cases are estimated at less than 1% of all cases. However, umbilical cutaneous endometriosis comprises 30%–40% of all cutaneous endometriosis.^2^

This is a rare presentation of de novo umbilical endometriosis that required resection and reconstruction of the anterior abdominal wall. The case was jointly treated with plastic surgery and gynecologic surgery, optimizing the patient for the best treatment outcome.

## CASE

**Fig. 1.**
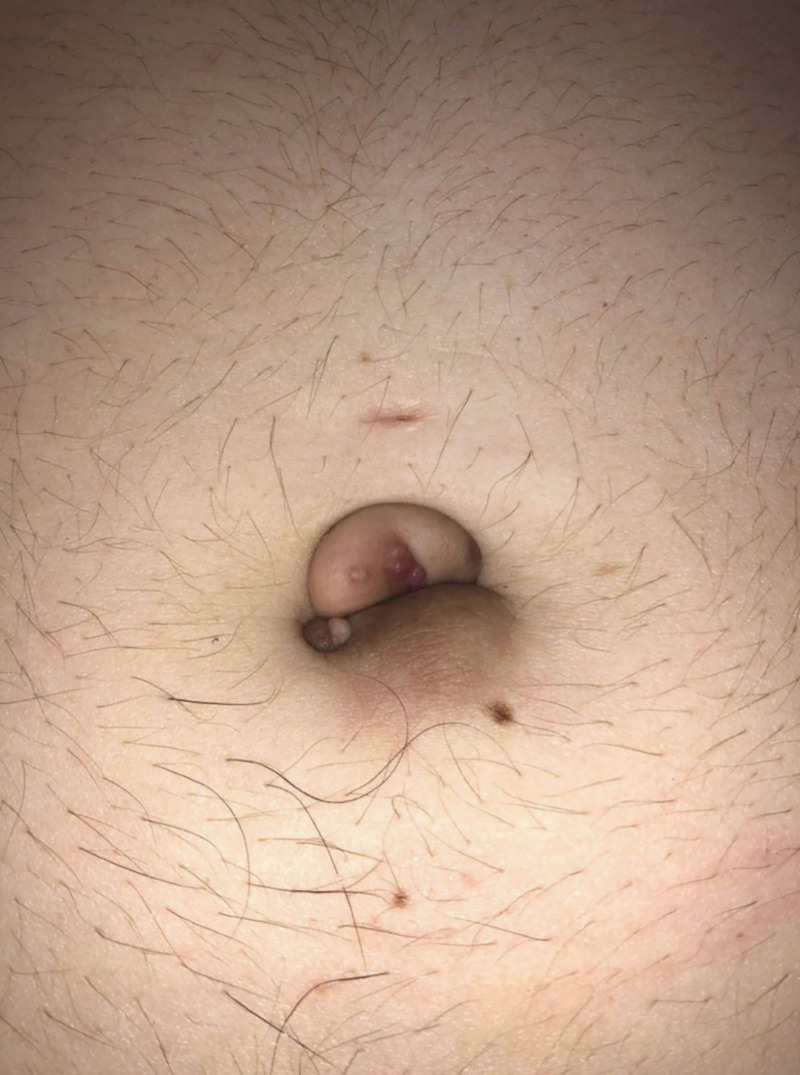
Initial physical examination showing 3-cm size endometrial implant on the superior aspect of the umbilicus and a 2-cm size cyst on the inferior aspect of the umbilicus.

A 36-year-old woman with a 10-year history of cutaneous endometriosis initially presented to minimally invasive gynecologic surgery (MIGS). She reported cyclic bleeding and pain from the umbilicus refractory to cauterization. On examination, she had a 3-cm size endometrial implant on the superior aspect of the umbilicus and a 2-cm size cyst on the inferior aspect of the umbilicus (Fig. 1) as well as signs concerning for a rectal nodule. She was also seen preoperatively by plastic surgery. Due to her supra umbilical hooding and laxity, a four-flap umbilicoplasty was proposed.

**Fig. 2.**
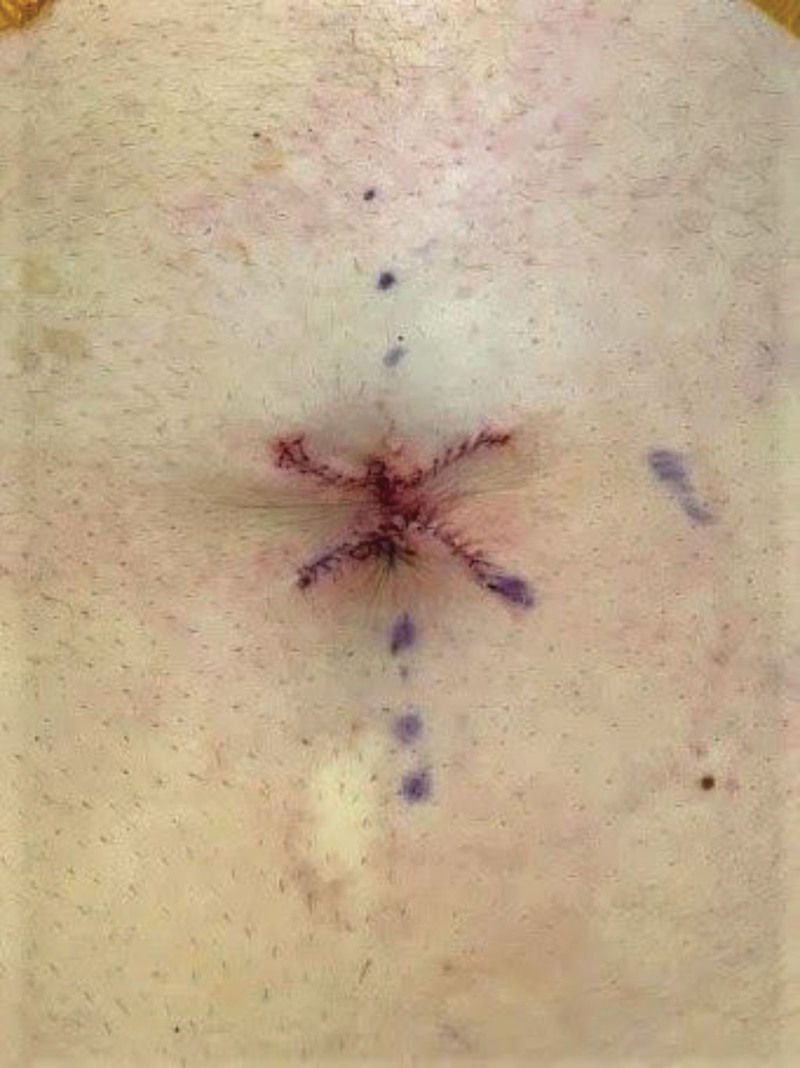
Four-flap umbilicoplasty immediately postoperative.

Joint surgery was performed with the MIGS and plastic surgery teams. (See Video [online], which displays the surgical technique of the four-flap umbilicoplasty in the treatment of umbilical endometriosis.) Plastic surgery performed a four-flap umbilicoplasty (Fig. 2). The umbilicus and mass were sharply excised using a 15 blade scalpel with 2 mm of normal tissue margin. The incision was taken down to the anterior rectus fascia until the specimen was fully excised. Laparoscopy was then performed by MIGS. Plastic surgery then resumed their portion of the case.

**Video 1.** Four-flap umbilicoplasty. Video 1 from “Joint Treatment of De Novo Umbilical Endometriosis with Plastic Surgery and Minimally Invasive Gynecologic Surgery”

The umbilical wound was noted to measure 3.0 × 3.2 cm. The four flaps were then created, each with a diameter of 2.2 cm and length of 1.5 cm. The lateral and inferior flaps were defatted away from the center of the wound. The length of defatting was 1 cm. The superior flap was left full thickness but mobilized off the underlying fascia to allow for advancement and to create supraumbilical hooding.

The inferior flap was sewn down to the anterior abdominal wall fascia using 3-0 PDS. The two lateral flaps were also sewn down using 3-0 PDS. The superior flap was then sewn down to the anterior rectus fascia. The flaps were closed using 3-0 Monocryl interrupted deep dermal sutures followed by a 5-0 fast-absorbing gut. The incisions were packed with Xeroform 2 × 2’s and Tegaderms to prevent stenosis.

The patient was discharged home the day of surgery. Pathology confirmed endometrial implant within the umbilical mass as well as on pelvic structures.

**Fig. 3.**
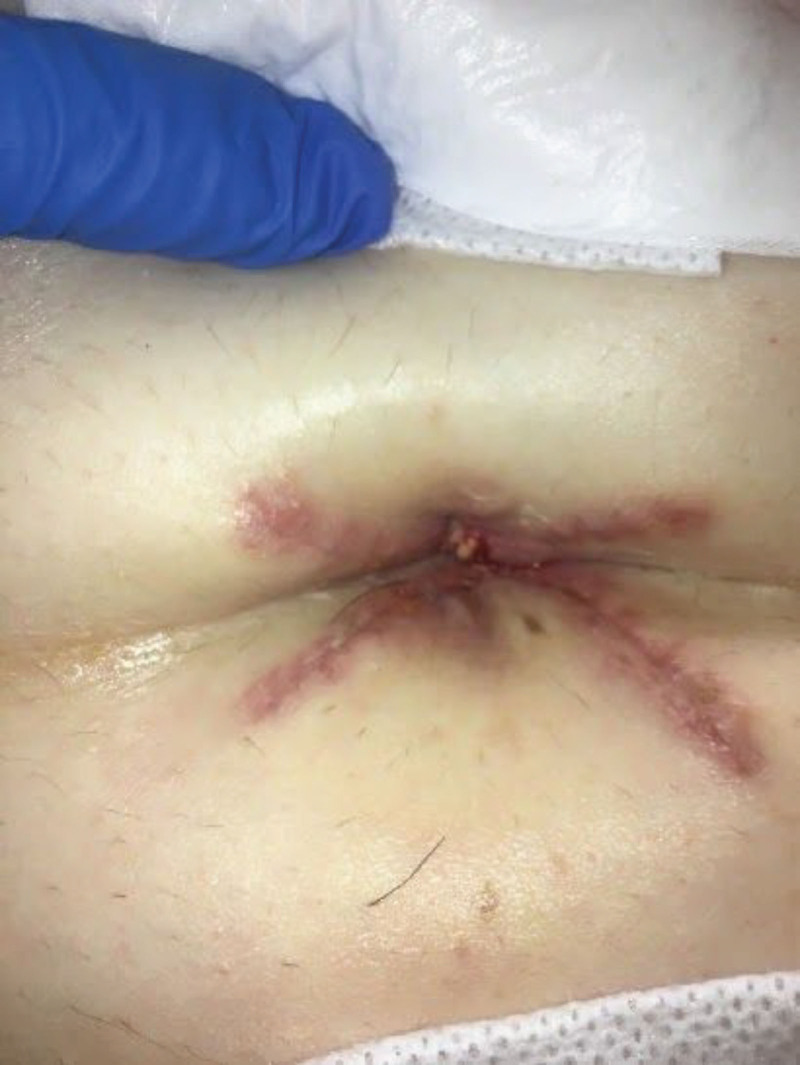
Four-flap umbilicoplasty three weeks postoperative. The patient was applying topical bacitracin twice daily to the umbilicus.

The patient presented for postoperative visit with plastic surgery three weeks after surgery stating she was happy with the result. She was applying topical bacitracin twice daily to the umbilicus as instructed (Fig. 3). She followed up again five weeks postoperatively and was noted to have excellent healing of umbilicoplasty.

She followed up in the MIGS office eight days postoperatively and reported menses starting two days after surgery, with no bleeding from the umbilical site. It was at that time she reported a desire to pursue pregnancy.

## DISCUSSION

**Fig. 4.**
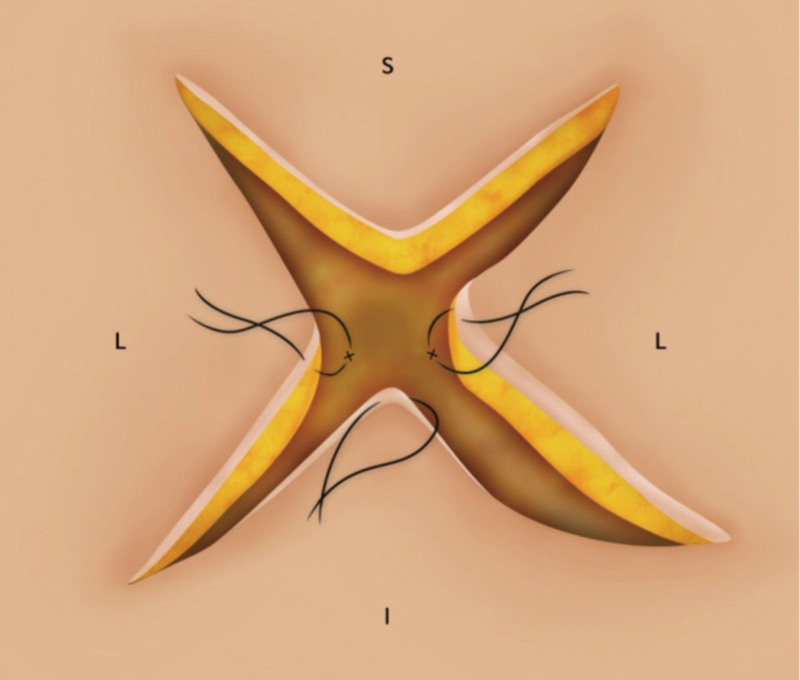
Surgical technique for the four-flap umbilicoplasty. Reprinted with permission from the *Archives of Plastic Surgery* 2015;42:351-355.

There is a range of published surgical techniques for reconstruction of the umbilicus.^3–8^ In this case, we document the novel use of the four-flap approach for the treatment of umbilical endometriosis^3,4,9^ (Fig. 4).

Given the size and location of her endometrial implants, this patient required full excision of the umbilicus to the anterior rectus fascia. The four-flap technique was ideal because it did not require any remaining umbilical tissue to create an aesthetically pleasing outcome. By preserving the thickness of the superior flap, this technique also recreated supraumbilical hooding, which allowed the patient to have a reconstruction that would resemble her preoperative umbilicus. The tissue immediately surrounding the umbilicus was healthy and free of endometrial implants. This well vascularized tissue improved healing and concisely recreated her umbilicus. The technique, given its circumferential symmetry, is also straightforward to learn and easy to perform.

A number of documented techniques, such as the dome procedure,^5^ diamond skin flap,^6^ rabbit head flap,^7^ and pumpkin teeth advancement flaps^8^ have also been documented as approaches to umbilical reconstruction. However, these techniques are either primarily used in a pediatric population, performed as a delayed procedure, or used in cases when a large vertical or horizontal incision traverses the umbilicus. Due to the minimally invasive approach used by the gynecologic team, we aimed to limit the incision on her abdominal wall.

Alternatively, the purse string and perforator flap methods have been documented techniques for umbilicoplasty in cases of umbilical endometriosis^4,10^ However these techniques can lead to stenosis of the umbilicus with a shallow scar. The four-flap technique leaves no visible scars, has a low complication rate, and is associated with high patient satisfaction.^11^

Although there are documented case reports of umbilical endometriosis, the team-based approach in the treatment of umbilical and pelvic endometriosis has not previously been published.^12–14^ There are a handful of published reports documenting interspecialty collaboration in the management of extraperitoneal sites of endometriosis, but of those, only a few show plastic surgery as a primary or consulting team.^1,15^

Plastic surgeons and other subspecialists should feel comfortable consulting colleagues in gynecology to jointly treat cases of endometriosis. This can improve patient outcome, reduce the total number of surgeries a patient undergoes, and preserve future fertility.^16^

## CONCLUSIONS

The use of a four-flap umbilicoplasty technique was a novel treatment approach for this patient with a rare de novo presentation of umbilical endometriosis. Plastic surgeons should consider this technique when presented with a case of umbilical endometriosis. Interspecialty collaboration to optimize patient outcomes was also a key aspect of her care. It is important to ensure that patients with endometriosis continue follow up with a gynecologist for continued management and to reduce the need for further corrective surgeries. The team-based approach utilized between minimally invasive gynecologic surgery and plastic surgery for this patient’s umbilical surgery proved to provide adequate treatment of her endometriosis with the added benefit of a cosmetically appealing outcome.
